# Long-term outcomes of daily oral vs. pulsed intravenous cyclophosphamide in a non-trial setting in ANCA-associated vasculitis

**DOI:** 10.1007/s10067-017-3944-7

**Published:** 2017-12-15

**Authors:** Jonathan La-Crette, Jeremy Royle, Peter C Lanyon, Alastair Ferraro, Amanda Butler, Fiona A Pearce

**Affiliations:** 1 Department of Rheumatology, Nottingham University Hospitals NHS Trust, Nottingham, UK; 2 Nottingham NHS Treatment Centre, Nottingham, UK; 3 Division of Rheumatology, Orthopaedics and Dermatology, University of Nottingham, Nottingham, UK; 4 Department of Renal Medicine, Nottingham University Hospitals NHS Trust, Nottingham, UK; 5 Division of Epidemiology and Public Health, B126, Clinical Sciences Building, City Hospital, University of Nottingham, Nottingham, NG5 1PB UK

**Keywords:** ANCA-associated vasculitis, Cyclophosphamide, Neutropenia

## Abstract

We aimed to compare risk of death, relapse, neutropenia and infection requiring hospital admission between unselected ANCA-associated vasculitis (AAV) patients according to whether cyclophosphamide induction was by daily oral (PO) or pulse intravenous (IV) route. We identified all newly diagnosed AAV patients treated with PO or IV cyclophosphamide between March 2007 and June 2013. We used Cox and logistic regression models to compare mortality, relapse and adverse events and adjusted these for age, renal function and other significant confounders. Fifty-seven patients received PO and 57 received IV cyclophosphamide. One-year survival was 86.0% in PO and 98.2% in IV patients; all-time adjusted hazard ratio (HR) for PO compared to that of IV cyclophosphamide was 1.8 (95% CI 0.3–10.6, *P* = 0.54). One-year relapse-free survival was 80.7% in PO compared to 87.3% in IV patients, all-time adjusted HR 3.8 (0.2–846, *P* = 0.37). During the first 12 months, neutropenia of ≤ 0.5 × 10^9^/L occurred in 9 (16%) PO and 0 (0%) IV cyclophosphamide patients (*P* = 0.003). The number of patients admitted with one or more infections was 16 (28%) in the PO group and 9 (16%) in the IV group, adjusted OR 2.2 (0.6–8.6, *P* = 0.23). We observed an increased risk of neutropenia, a trend towards increased risk of death and an admission with infection with PO cyclophosphamide. This adds certainty to previous studies, indicating that PO administration induces greater marrow toxicity. Infection-related admissions within 12 months of starting cyclophosphamide were higher than those in clinical trials, possibly reflecting the unselected nature of this cohort.

## Introduction

Anti-neutrophil cytoplasmic antibody (ANCA)-associated vasculitis (AAV) is the commonest life-threatening vasculitis of adults [1], and is characterised by inflammation of small and medium blood vessels, and associated with detectable ANCAs in serum. It comprises three overlapping conditions: granulomatosis with polyangiitis (formerly known as Wegener’s granulomatosis), microscopic polyangiitis and eosinophilic granulomatosis with polyangiitis (formerly known as Churg-Strauss syndrome). National and international guidelines recommend initial remission induction treatment with cyclophosphamide [2, 3].

Cyclophosphamide is given either by daily oral (PO) or pulsed intravenous (IV) administration. The pulsed IV route is considered to have less toxicity; and CYCLOPS, an international, multi-centre clinical trial (*n* = 149) comparing the two regimens, demonstrated that both treatment routes were equally efficacious. Pulsed IV therapy had fewer side effects including leukopenia but is reported to have a higher relapse rate than the continuous oral route; however, long-term follow-up showed no difference in mortality or renal function at 5 years with either route [4, 5].

In many centres in the UK, people with AAV who present to rheumatology receive IV therapy and those who present to nephrology receive PO therapy [6]. There has been no comparison of the outcomes in routine clinical practice according to treatment route. This is relevant because patients with AAV in randomised trials are unlikely to be representative of all patients with AAV [7]. It is important to evaluate any effect of this variation in treatment route, not least because delivering day-case IV therapy has significantly higher healthcare costs.

Observational studies are an increasingly important approach for studying health outcomes in rare diseases [8]. In Nottingham, we previously identified a complete incidence cohort of AAV patients (March 2007–June 2013), with the treating specialty (rheumatology or nephrology) usually determined by the presence or absence of significant renal involvement. We aimed to compare outcomes of death, relapse, neutropenia, and infection requiring hospital admission between the two treatment groups in this unselected cohort which represents real-life clinical practice.

## Methods

All patients diagnosed with AAV at Nottingham University Hospitals NHS Trust between March 2007 and June 2013 have previously been identified by multiple case ascertainment strategies as part of an institutional audit [9]. In this study, we included all incident cases diagnosed between March 2007 and June 2013 and treated with cyclophosphamide. Diagnosis of AAV was classified according to the EMA criteria [10]. Retrospective case note analysis was undertaken to ascertain route of administration and baseline characteristics known to be associated with outcomes [11–14] including age, sex, baseline creatinine and eGFR, haemoglobin and white cell count, documented organ system involvement, ANCA status (anti-PR3, anti-MPO, negative), diagnosis, renal involvement and plasma exchange. Patients were followed up from time of diagnosis to their death or most recent hospital assessment. Outcomes were as follows: time to death, time to end stage renal failure, time to first relapse and hospital admissions due to infection or neutropenic sepsis in the first year after diagnosis. Occurrence of end stage renal failure (ESRF), diabetes, venous thromboembolism (VTE), cardiovascular event (myocardial infarction (MI) or stroke) and cancer during follow-up was also collected. Age was categorised into four groups: (i) < 50, (ii) 50 < 60, (iii) 60 < 70, (iv) ≥ 70 years and eGFR into bands of (i) > 60 ml/min/1.73m^2^, (ii) 30–60, (iii) < 30 but not on dialysis, (iv) requiring dialysis within 1 month of diagnosis.

Differences in mortality and time to relapse were assessed using Kaplan-Meier methods and Cox regression. Differences between treatment routes in neutropenia and infections requiring admission within the first year after diagnosis were analysed by Fisher’s exact test and logistic regression. Regression models are reported as crude (unadjusted) estimates and estimates adjusted for age category, sex, eGFR category and ANCA type which were included as a priori confounders. Data were analysed using the Stata 14 statistical software (StataCorp LP, College Station, TX, USA).

### Ethics

This study was approved by the Nottingham University Hospitals NHS Trust clinical audit department, as a service evaluation component of an audit (project number 13-037C). Informed patient consent was not required for this study.

### Reporting guidelines

This study has been reported following the Strengthening the Reporting of Observational Studies in Epidemiology (STROBE) guidelines [15].

## Results

One hundred fourteen patients started cyclophosphamide for a new diagnosis of AAV during the study period and were included in the analysis: 57 patients received PO cyclophosphamide and 57 patients received IV cyclophosphamide. Equal numbers occurred in each group by chance. Overall mean follow-up was 4.8 years: 4.4 (standard deviation [SD] 2.8) years in the PO and 5.3 (SD 2.6) years in the IV group.

### Baseline characteristics

Baseline characteristics and clinical features were significantly different between the two groups (Table 1), reflecting the clinical differences influencing whether patients would present to nephrology or rheumatology. The patients who received PO cyclophosphamide were older, had more renal involvement with higher creatinine and were more likely to have MPO-ANCA.

**Table 1:** Characteristics and treatment of patients receiving PO and IV cyclophosphamide

	PO cyclophosphamide group *N* (%)	IV cyclophosphamide group *N* (%)	*P* value for difference between groups
Age (median, IQR)	72 (65–78)	55 (44–68)	< 0.0001*
Male	28 (49%)	31 (54%)	0.57
PR3-ANCA	22 (39%)	35 (61%)	0.02*
MPO-ANCA	32 (56%)	17 (30%)	
ANCA negative	3 (5%)	5 (9%)	
Renal involvement	56 (98%)	26 (46%)	< 0.001*
ENT involvement	9 (16%)	33 (58%)	< 0.001*
Neurological involvement	2 (4%)	19 (33%)	< 0.001*
Eye involvement	0	5 (9%)	0.02*
Abdominal involvement	0	6 (11%)	0.01*
General/systemic involvement	18 (32%)	27 (47%)	0.09
Cutaneous involvement	5 (9%)	9 (16%)	0.25
Pulmonary involvement	23 (40%)	25 (46%)	0.57
Cardiovascular involvement	2 (4%)	2 (4%)	1.0
Serum creatinine	295 (158–618)	80 (68–100)	< 0.0001*
Plasma exchange	24 (42%)	8 (14%)	0.001*
Refractory disease	1 (2%)	2 (4%)	0.5
Follow-up time (years)	4.0 (2.6–6.7)	5.1 (3.2–7.6)	0.10
Prednisolone dose at diagnosis	45 (30–60)	60 (42.5–60)	0.06
Prednisolone dose at 3 months	15 (15–20)	15 (10–20)	0.15
Prednisolone dose at 6 months	10 (7.5–10)	7.5 (5–10)	0.10
Prednisolone dose at 1 year	5 (5–7.5)	5 (2.5–7.5)	0.15
Creatinine at 5 years	142 (76–206)	86 (76–107)	< 0.0001*

*P value significant at 95% confidence level

### Treatment

Prednisolone dose at diagnosis was median 45 mg (IQR 30–60 mg) in the PO cyclophosphamide group and 60 mg (42.5—60 mg) in the IV cyclophosphamide group. By 1 year, both groups had median prednisolone dose of 5 mg (Table 1) in patients who had not relapsed. Three patients had cyclophosphamide-refractory disease at 3 months: one in the PO cyclophosphamide group and two in the IV cyclophosphamide group. Thirty-two patients received plasma exchange: 24 in the PO cyclophosphamide group and 8 in the IV cyclophosphamide group.

### Outcomes

Results are shown in Table 2.

**Table 2:** Outcomes of patients receiving PO cyclophosphamide compared to IV cyclophosphamide

	PO cyclophosphamide group *n* (%)	IV cyclophosphamide group *n* (%)	Unadjusted HR/OR for PO cyclophosphamide	Adjusted HR/OR for PO cyclophosphamide^a^	*P* value (for adjusted HR/OR)
Mortality	17 (30%)	5 (9%)	3.9 (1.4–10.4)	1.9 (0.3–12.0)	*P* = 0.47
Relapse	14 (25%)	24 (42%)	0.5 (0.3–1.0)	1.0 (0.4–2.6)	*P* = 1.00
Neutropenia	9 (16%)	0 (0%)	–	–	*P* = 0.003*
Infection requiring admission	16 (28%)	9 (16%)	2.0 (0.8–5.0)	2.2 (0.6–8.7)	*P* = 0.24

Hazard ratio (HR) for mortality and relapse rates during the whole follow-up period was calculated using Cox regression. Odds ratio (OR) for neutropenia or infection requiring hospital admission within 1 year after diagnosis was calculated using logistic regression

*P value from Fisher’s exact test (unadjusted for confounders)

^a^Adjusted for age category, sex, eGFR category and ANCA type

### Mortality/survival

Overall, 22 patients died in 553 patient-years of follow-up (mortality rate of 39.8 per thousand person-years). One-year survival was 86.0% in PO and 98.2% in IV patients. Survival to the end of the follow-up period was 70% in PO and 91% in IV patients. Unadjusted hazard ratio (HR) for the whole follow-up period for PO compared to IV cyclophosphamide was 3.9 (95% CI 1.4–10.4), and adjusted HR was 1.9 (95% CI 0.3–12.0, *P* = 0.47).

### Relapse

One-year relapse rate was 4 (7%) in PO and 6 (11%) in IV patients; relapse rate to the end of the follow-up period was 14 (25%) in PO and 24 (42%) in IV patients. Unadjusted hazard ratio (HR) for the whole follow-up period for PO compared to that of IV cyclophosphamide was 0.5 (95% CI 0.3–1.0), and adjusted HR was 1.0 (95% CI 0.4–2.6, *P* = 1.00).

### Neutropenia

During the first 12 months after initiating treatment, documented neutropenia of ≤ 0.5 × 10^9^/L occurred in 9 (16%) PO and 0 (0%) IV patients (*P* = 0.003). It was not possible to calculate an odds ratio because the outcome did not occur in IV patients. The lowest documented neutrophil count was 0.1 in the PO group and 0.94 in the IV group. Five patients in the PO group were treated for neutropenic sepsis with no recorded cases in the IV group.

### Infection

Overall, 25 (22%) patients were admitted to hospital with ≥ 1 infection during 12 months after initiation treatment: 16 (28%) with PO and 9 (16%) with IV cyclophosphamide. Unadjusted OR for admission with infection in PO compared to that of IV cyclophosphamide was 2.0 (95% CI 0.8–5.0), and adjusted OR was 2.2 (95% CI 0.6–8.7, *P* = 0.24).

### Other outcomes

Overall, ESRF occurred in 20 (18%); incident diabetes, in 14 (13%); VTE, in 8 (7%); MI, in 8 (7%); stroke, in 8 (7%) and any cancer, in 9 (8%).

### Cause of death

Cause of death is recorded for 18 of the 22 deaths. Of the 9 deaths in the first year, 5 (56%) were due to infection (4 pneumonia, 1 *E*. *coli* sepsis), 1 progressive lung fibrosis, 1 lung cancer, 1 retroperitoneal haemorrhage and 1 unknown. Of the 13 deaths after the first year, 7 (54%) were due to infection (3 pneumonia, 2 infective exacerbation of bronchiectasis, 2 systemic sepsis with source not specified) 1 MI, 1 old age, 1 renal failure and 3 unknown.

## Discussion

Patients who received PO and IV cyclophosphamide were significantly different at baseline, reflecting differences in the AAV phenotype between patients presenting to rheumatology and nephrology departments. Patients presenting to nephrology were older and had almost universal renal involvement, both of which are associated with worse outcomes. Although there were stark differences in crude outcomes, once adjusted for age, sex and renal function, there were no significant differences between PO and IV cyclophosphamide in survival or relapse rates. PO cyclophosphamide was associated with a significant risk of neutropenia compared to IV cyclophosphamide. Although not significant, there was a trend towards an increased risk of admission with infection in the PO group.

The main strengths of our study are long follow-up times (mean 4.8 years) and that data capture-recapture techniques suggested our case identification technique resulted in near complete case capture [9], resulting in an unselected cohort likely to be representative of real-life outcomes in routine clinical practice. This suggests our results may be more generalizable to the whole population of AAV patients than those of clinical trials, whose participants represent selected patients and have been shown to differ from patients within observational cohorts of AAV [7].

Our study is limited by its observational nature. However, observational studies of outcome are important in rare diseases and can help to increase the level of certainty about specific clinical questions. The main limitation is the risk of confounding; treatment route was likely to have been determined by the baseline characteristics, with older patients and those with renal dysfunction (both risk factors for poor outcomes) more likely to be treated by nephrology and therefore receive PO cyclophosphamide. The sample size was too small to use propensity score methods to adjust for this; we therefore adjusted the analysis for the known confounders (age category, sex, eGFR category and ANCA type). We are unlikely to have been completely successful in adjusting; however, despite the theoretical limitations, our adjustment led to attenuation of the crude differences in treatment effects in the adjusted models.

Whereas in the CYCLOPS study, patients received a median cyclophosphamide dose of 8.58 g IV and 18.05 g PO [4] and our protocol aims to deliver similar cyclophosphamide doses of ~ 10 g to both PO (2 mg/kg for 3 months) and IV (15 mg/kg for 10 cycles over 6 months) groups. However, our remission maintenance protocol mimics the CYCLOPS protocol using azathioprine as the first-line maintenance agent. In light of this, but keeping in mind that we are comparing a randomised controlled trial where baseline differences between the treatment groups are evened out by randomisation to our cohort where there are very different baseline characteristics in the treatment groups, it is interesting to compare our results to the CYCLOPS trial. We observed a similar non-significant higher mortality in the PO arm but not a greater risk of relapse in the IV arm [4, 5]. It is tempting to speculate that this may be linked to differing cyclophosphamide doses used in the two scenarios. As in CYCLOPS, we saw no statistically significant difference in survival or renal function at the end of the study period.

Contrasting with CYCLOPS, we observed an overall rate of admission with infection during the first year of 22% (28% PO, 16% IV). Only a few admissions were at times of frank neutropenia, and the steroid doses in our cohort were marginally lower than those of the CYCLOPS protocol. The average age of patients in our IV treatment cohort was similar to that of the CYCLOPS cohort; however, our PO cohort was significantly older; advancing age is a risk factor for hospitalisation with infection. Additionally, a quarter of our cohort was made up of patients who would have been excluded by the CYCLOPS trial protocol, e.g. life-threatening disease or creatinine > 500 μmol/L.

The finding of differences between patients with AAV presenting to nephrology or rheumatology services is consistent with that of a recent paper from Ireland describing patients with AAV presenting to Galway University Hospitals from June 2002 to July 2011 [16]. They reported on 45 patients, and like our study found that the patients presenting to nephrology were significantly older and had higher creatinine than those presenting to rheumatology. Additionally, they reported baseline Birmingham Vasculitis Activity Scores (BVAS) and found these to be significantly higher among patients presenting to nephrology compared to rheumatology.

The main cause of mortality in our study was infection, accounting for 56% of deaths in the first year after diagnosis and 54% of later deaths, with none recorded as due to active vasculitis, suggesting that in our cohort, the increased risk of infection extends beyond the first year. This does not completely align with previous studies of cause of death where in the first year after diagnosis, the most frequent causes were infection (32–48% of deaths) then active vasculitis (18–19% of deaths), with later deaths most frequently due to cardiovascular disease, malignancy and renal failure [14, 17].

In conclusion, PO cyclophosphamide was associated with an increased risk of neutropenia, and a trend towards increased risk of death, and admission with infection with PO cyclophosphamide. This adds to the level of certainty that PO cyclophosphamide is associated with greater marrow toxicity that was suggested in the CYCLOPS study results and a previous meta-analysis that demonstrated increased risk of leukopenia with this route [18]. This is important, because leukopenia is known to be associated with increased mortality and infection, even though this was not statistically demonstrated in the cohort reported here [19, 20].

Of note in our study, in the setting of routine clinic practice, over 20% of the patients were admitted with infection in the first year. It remains to be seen if further developments in the management of ANCA vasculitis (e.g. steroid sparing protocols) can impact upon this, but it highlights the need for ongoing clinical vigilance during the treatment of this complex disease.
